# Chirality Effects in Peptide Assembly Structures

**DOI:** 10.3389/fbioe.2021.703004

**Published:** 2021-06-22

**Authors:** Yongfang Zheng, Kejing Mao, Shixian Chen, Hu Zhu

**Affiliations:** Engineering Research Center of Industrial Biocatalysis, Fujian Province Universities, Fujian Provincial Key Laboratory of Advanced Materials Oriented Chemical Engineering, Fujian Provincial Key Laboratory of Polymer Materials, College of Chemistry and Materials Science, Fujian Normal University, Fuzhou, China

**Keywords:** peptide self-assembly, chirality effects, chirality switching, co-assembly, bioactivity

## Abstract

Peptide assembly structures have been widely exploited in fabricating biomaterials that are promising for medical applications. Peptides can self-organize into various highly ordered supramolecular architectures, such as nanofibril, nanobelt, nanotube, nanowire, and vesicle. Detailed studies of the molecular mechanism by which these versatile building blocks assemble can guide the design of peptide architectures with desired structure and functionality. It has been revealed that peptide assembly structures are highly sequence-dependent and sensitive to amino acid composition, the chirality of peptide and amino acid residues, and external factors, such as solvent, pH, and temperature. This mini-review focuses on the regulatory effects of chirality alteration on the structure and bioactivity of linear and cyclic peptide assemblies. In addition, chiral self-sorting and co-assembly of racemic peptide mixtures were discussed.

## Introduction

Molecular self-assembly refers to the process in which basic structural units spontaneously form stable and ordered structures through non-covalent bond interactions, such as hydrophobic interactions, hydrogen bonds, van der Waals interactions, and electrostatic interactions. Self-assembly is ubiquitous and plays a vital role in biological systems: phospholipids form biological membranes through self-assembling; DNA strands form double helix structures through hydrogen bonds; proteins fold into correct structures; protein misfolding and aggregation lead to neurodegenerative diseases. Meanwhile, self-assembly is a very important “bottom-up” strategy for constructing supramolecular materials. Many biomolecules have been demonstrated to self-organize into well-ordered nanostructures. Among them, peptides have become widely used building blocks in constructing supramolecular architectures due to their easy synthesis and modification, good biocompatibility, biodegradability, and easy availability for “bottom-up” fabrication.

Although a lot of progresses have been made in studying the molecular mechanism of peptide assembly, it remains a challenge to accurately regulate the assembly structure of peptides to achieve pre-designed structure and function. Chirality, an inherent property of peptides, has been recognized as a vital factor that can exert essential impacts on peptide assembly structures. Since the thalidomide incident in the 1950s, the importance of molecular chirality has been recognized. Therefore, in the development of peptide biomaterials, the chirality of peptides and amino acid residues is an important factor that has been taken into consideration by researchers. It has been suggested that amino acids and peptides with different chirality have different effects on protein adsorption (Wang et al., 2011), peptide assembly (Qing et al., 2014; Hou et al., 2020), and cell behaviors (Yao et al., 2013; Ma et al., 2020). With the rapid development of supramolecular chemistry and the promising application of peptide assembly structures in the field of biomedicine, the influence of molecular chirality on the structure and function of peptide assemblies has been a key and hot research field. In this mini review, we focus on the chirality effects in peptide assemblies. We summarized the recent advances in the structurally regulatory effects of chirality alteration on linear and cyclic peptide assemblies. Chiral self-sorting and co-assembly of mixed racemic peptides were discussed. Besides, we analyzed the influence of the chirality effects on the biological activities of peptide assembly structures.

## Manuscript Formatting

### The Effects of Chirality Switching on Assembly Structures of Linear Peptides

Introducing *D*-amino acids into *L*-peptides can distort their main chains and destroy their original secondary structure. The effects of *D*-amino acid substitution on α-helix structure have been studied for collagen-mimicking peptides. It was suggested that *D*-amino acid substitution could break α-helix structure by inducing kink structure, and the helix-destabilizing ability was highly dependent on the steric hindrance of amino acid side chains (Imperiali et al., 1992; Krause et al., 2000; Punitha et al., 2009). Chirality alteration of amino acid residues can break the secondary structure of peptides, thereby destroying their assembly structures. Peptide EAK16 self-assembled into nanofibers, while E*^*D*^*AK16 and *^*D*^*EA*^*D*^*K16 could not undergo self-organization to form a well-ordered structure, as *D*-amino acid incorporation drastically disrupted its β-sheet structure (Luo et al., 2011). We studied the effects of chirality switching of a single amino acid residue at different positions and with various side chain moieties on peptide assembly structure using scanning tunneling microscope (STM) that is a very useful tool in studying peptide assembly structures at the single-molecule level (Yu et al., 2018, 2020; Zheng et al., 2019a,b). The molecular observations revealed that chirality switching of single amino acid was able to break the β-sheet structure and destabilize the surface-mediated peptide assemblies, and this disturbance effect was site-dependent and positively correlated with the steric hindrance of amino acid side chains (Zheng et al., 2019b). The above results indicate that heterochirality leads to weakening self-assembly propensity for some sequences.

**FIGURE 1:**
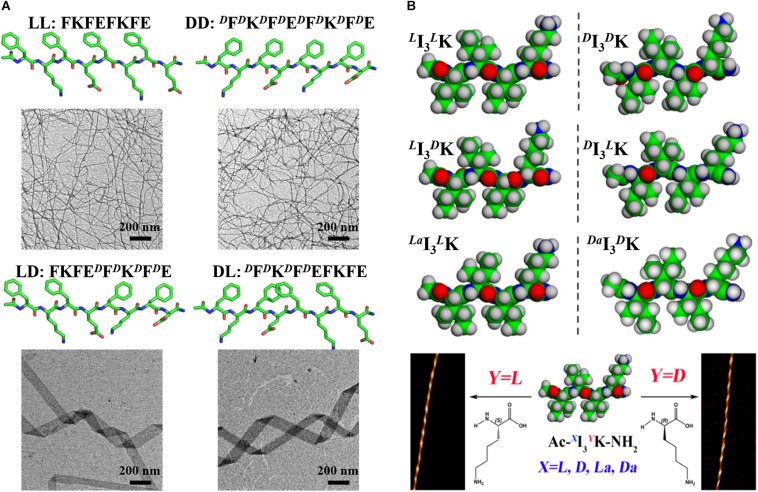
The effects of amino acid chirality alteration on peptide assembly structure. **(A)** Amino acid chirality alteration drastically changes the morphology of assembly structure of the peptide Ac-(FKFE)_2_-NH_2_. Reproduced with permission from Clover et al. (2020). Copyright 2020 American Chemical Society. **(B)** The handedness of fibers formed by I_3_K is controlled by the chirality of the C-terminal hydrophilic Lys. Reproduced with permission from Wang M. et al. (2017). Copyright 2017 American Chemical Society.

In contrast, for some sequences, they can still form ordered nanostructures after *D*-amino acid incorporation, just with the morphology and handedness of their assembly structures being changed. The effects of amino acid chirality alteration on the assembly structures of diphenylalanine (FF) and its derivatives have been investigated. It was suggested that replacing one Phe of FF with its *D*-enantiomer preserved its ability to self-assemble into nanotubes and the heterochirality made the nanotubes more homogeneous and stable (Kralj et al., 2020), while switching the chirality of one Phe of FF derivatives, such as Fmoc-FF-Fmoc and Nap-FF, changed the morphology of their assembly structures (McAulay et al., 2019; Gil et al., 2020). The Rudra group explored the effects of multiple and consecutive amino acid chiral mutations on the assembly structure of peptide Ac-(FKFE)_2_-NH_2_ (Clover et al., 2020). The results showed that the heterochiral analogs of the model peptide, composed of two FKFE repeat motifs with opposite chirality, self-assembled into helical tapes with a width of 108 ± 55 nm and a pitch of 900–1200 nm. As shown in Figure 1A, the dimension and pitch greatly exceeded those of the fibers formed by the homochiral analogs. According to the results from molecular dynamics simulation, the authors postulated that chirality alteration caused a kink structure between the two repeat motifs and introduced an internal strain, which countered the natural twist of the β-sheet structure and made it flattening, resulting in a much longer pitch. The supramolecular chirality of peptide assemblies can be regulated by the chirality of single amino acid residue. Xu and coworkers designed three pairs of enantiomeric peptides (*^*L*^*I_3_*^*L*^*K and *^*D*^*I_3_*^*D*^*K, *^*L*^*I_3_*^*D*^*K and *^*D*^*I_3_*^*L*^*K, and *^*La*^*I_3_*^*L*^*K and *^*Da*^*I_3_*^*Da*^*K) by altering the chirality of α-carbon and side-chain β-carbon atoms of the short amphiphilic peptide I_3_K. It was shown that all the peptides self-assembled into twisted fibers, just with different twisted handedness which was found to be controlled by the chirality of the C-terminal hydrophilic Lys head (Figure 1B) (Wang M. et al., 2017). The assembly structures of fatty chain-modified dialanine with homochirality and heterochirality have been characterized, showing that the handedness of the fibers was dependent on the chirality of the terminal alanine (Fu et al., 2013; Li et al., 2013). These results indicate the significance of the chirality of terminal amino acid residues in determining supramolecular chirality. On the contrary, Feng and coworkers studied the assembly structures of dipeptides derivatives by connecting two dipeptide arms (FF, AA, FA, and AF) with different chirality to para-disubstituted phenyl group, and found that the supramolecular chirality was only determined by the amino acid residue adjacent to the benzene core and irrespective of the chirality of C-terminal amino acid residue (Qin et al., 2021). In addition, the handedness of the nanofibers formed by bola-type dipeptides (AF) was dictated by the phenylalanine residue, not by the terminal amino acid residues (Zheng et al., 2020).

Unexpectedly, some sequences showed a divergent trend that heterochirality made them prone to self-organization. It has been demonstrated that chirality conversion of the first N-terminal amino acid residue of the non-assembling *L*-peptides VFF, FFV, and LFF from *L* to *D* enables them to form β-sheet structure and self-assemble into hydrogels (Marchesan et al., 2012a,b). Taking LFF as an example, the authors investigated the molecular mechanism by combining molecular modeling and X-ray diffraction (XRD), and found that *^*D*^*LFF formed a phenylalanine zipper structure that promoted its self-assembling, and it was not accessible for the homochiral LFF due to steric hindrance from the side chain of the *L*-leucine (Marchesan et al., 2012b). In 2018, Marchesan et al. designed a series of heterochiral tripeptides *^*L*^*Phe-*^*D*^*X-*^*L*^*Phe (*^*D*^*X stands for hydrophobic amino acids in *D* configuration) (Garcia et al., 2018). They speculated that this alternating arrangement of *L*- and *D*-amino acid could make all the hydrophobic amino acid side chains located on one side of the main chain of the peptide. As a result, the side chains function as a hydrophobic part, and the main chains act as a hydrophilic part, which gives the heterochiral tripeptides amphiphilicity and facilitates their self-assembling and the formation of hydrogels. The experimental results were consistent with the prediction showing that the heterochiral tripeptides self-assembled into fibrillar hydrogels, while most homochiral tripeptides formed amorphous aggregates.

The overall configuration of peptide has a significant impact on its self-assembled structure. *L*-peptide and *D*-peptide can form fibers with different handedness (Koga et al., 2005). In general, *L*-peptide forms left-handed helical structure, while its enantiomer *D*-peptide forms right-handed helical structure. Nevertheless, there are many exceptions. For example, peptide ILQINS, the key segment of hen egg white lysozyme, and serum amyloid A (SAA) truncated peptides SAA_1–12_ and SAA_2–12_ formed unexpected right-handed twisted fibers (Rubin et al., 2010; Lara et al., 2014).

### The Effects of Chirality Switching on Assembly Structures of Cyclic Peptides

Compared with linear peptides, literature reports on the chirality effects in assembly structures of cyclic peptides are far fewer. There are a few reports on the chirality effects on cyclic dipeptides, the simplest cyclic peptide (Govindaraju et al., 2011; Jeziorna et al., 2015). As in linear peptides, chirality switching of amino acid residues can regulate morphology and macroscopic propensities of cyclic peptide assemblies. For example, cyclo-(YA) formed nanotubes and nanowires, while cyclo-(Y*^*D*^*A) formed microtubes (Jeziorna et al., 2015). The 2D mesosheets formed by cyclo-(Phg-*^*D*^*Phg) are more thermodynamically stable than the mesosheets formed by cyclo-(Phg-Phg) (Govindaraju et al., 2011). For cyclic peptides with more than two amino acid residues that can self-organize into ordered structures, most of them are composed of alternating *D*- and *L*-amino acid residues. In 1974, according to theoretical analysis, Santis et al. (1974) predicted that cyclic peptides comprised of an even number of alternating *D*- and *L*-amino acid residues with all side chains pointing to the outside of the ring would stack through main chain–main chain hydrogen bonds. Therefore, the self-assembled cyclic peptides almost take the arrangement of alternating *D*- and *L*-amino acid residues and exclusively self-assemble into nanotubes (Insua and Montenegro, 2020; Song et al., 2020). In 2018, Li et al. introduced an in-tether chiral group to liner peptides and designed a type of cyclized helical peptides that only consist of *L*-amino acid residues. It has been revealed that this kind of peptide can self-assemble into well-ordered nanostructures, and their assembly behaviors can be governed by the in-tether chiral center (Hu et al., 2018, 2020). For unmodified *L*-cyclic peptides, more explorations are needed to clarify their assembly propensity and the effects of chirality alteration on their assembly structures, which can not only broaden the building blocks of peptide assemblies, but also contribute to understanding the chirality effects in assembly structures of peptides with limited rotation and structural rigidity.

### Chiral Self-Sorting and Co-assembly of Mixed Enantiomeric Peptides

**FIGURE 2:**
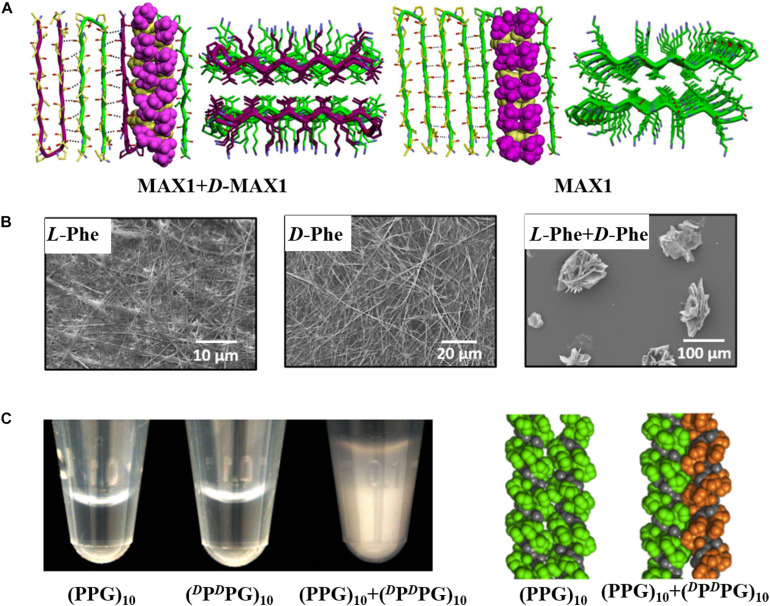
Co-assembly structures of racemic peptides and amino acids. **(A)** Model of MAX1/*D*-MAX1 in their co-assembled structure and model of pure MAX1 in its self-assembled structure. Reproduced with permission from Nagy-Smith et al. (2017). Copyright 2017 American Chemical Society. **(B)** The co-assembly structure of *L*-Phe and *D*-Phe is totally different from the self-assembly structure of the pure enantiomers. Reproduced with permission from Bera et al. (2020). Copyright 2020 American Chemical Society. **(C)** Mixing (PPG)_10_ and (*^*D*^*P*^*D*^*PG)_10_ drastically lowered the solubility and packing model of (PPG)_10_ self-assembled structure and (PPG)_10_/(*^*D*^*P*^*D*^*PG)_10_ co-assembled structure. Reproduced with permission from Xu et al. (2013). Copyright 2013 American Chemical Society.

In recent years, researchers have paid more and more attention to the assembly structures of racemic peptide mixtures. Chiral selectivity is ubiquitous in nature, so it is expected that proteins and peptides tend to prefer homochiral molecular interactions. Studies of *L*- and *D*-stereoisomers of amyloid peptides, such as a 22-residue segment of β_2_-microglobulin and β-amyloid peptide (Aβ40), showed that amyloid fibril formation was stereospecific: *L*-enantiomer was deposited on *L*-seeds, and enantiomers did not cross-react with each other (Esler et al., 1999; Wadai et al., 2005; Gupta et al., 2020). However, it has been demonstrated that mixing enantiomers can change the kinetics, morphology, and mechanical properties of peptide self-assemblies (Nagy-Smith et al., 2017; McAulay et al., 2019; Bera et al., 2020; Qin et al., 2021). For example, the racemic mixture of Fmoc monosubstituted cyclo-(EE) and its *D*-analog formed quickly recoverable thixotropic hydrogel with a significantly shortened thixotropic recovery time compared with the hydrogels formed by either enantiomer alone (Wang L. et al., 2017). In addition, the racemic gel formed by diphenylalanine-based derivative enantiomers was more mechanically robust than the gels formed by either pure enantiomer (Qin et al., 2021). On the contrary, He and coworkers got the opposite results showing that the hydrogel formed by a racemic mixture of ferrocene-diphenylalanine (Fc-FF) was mechanically weaker than the enantiopure hydrogels (Zhang et al., 2020). These differences in assembly behaviors between racemic mixtures and the pure enantiomers suggest that racemic mixtures may form a distinct new structure. As early as 1953, it was predicted by Pauling and Corey (1953) in their pioneering study that equimolar mixtures of two enantiomeric peptides were observed to pack into rippled β-sheet structure which contained alternating *L*- and *D*-sequences and revisited recently by a number of investigations (Nagy et al., 2011; Swanekamp et al., 2012; Kar et al., 2014; Nagy-Smith et al., 2017; Jeena et al., 2019). The Nilsson group demonstrated that equimolar mixtures of *L*-Ac-(FKFE)_2_-NH_2_ and *D*-Ac-(FKFE)_2_-NH_2_ did not self-sort into enantiomerically pure helical ribbons, but co-assembled into flat nanoribbons with alternating *L*- and *D*-enantiomer in “rippled β-sheet” orientation (Swanekamp et al., 2012). In 2011, the Schneider group found that a racemic mixture of β-hairpin peptides MAX1 and *D*-MAX1 formed a fibrillar hydrogel with enhanced mechanical rigidity compared with pure enantiomeric gels formed from either peptide alone (Nagy et al., 2011). In 2017, this group explored the molecular basis for the puzzling enhanced mechanical rigidity by using an arsenal of techniques, finding that it was due to the maximized inter-residue hydrophobic interactions achieved by the staggered arrangement of residues in the rippled β-sheet structure, as shown in Figure 2A (Nagy-Smith et al., 2017). Amyloid peptide polyglutamine (polyQ) was found to lack stereochemical restriction in seeded elongation of its amyloid fibers, as the fibrils formed by D-polyQ could efficiently seed the aggregation of L-polyQ monomers *in vitro*, and vice versa (Kar et al., 2014). The authors postulated that “rippled β-sheet” interfaces existed between seed fibrils and deposited monomers with opposite chirality. Besides, enantiomeric amino acids can co-assemble into nanostructures with enhanced mechanical rigidity. The Gazit group explored the assembly behaviors of the mixed aromatic amino acid enantiomers (Phe and Trp) via diverse experimental techniques (Bera et al., 2020). It was revealed that enantiomeric amino acids co-assembled into nanostructures with different morphology and kinetics compared with the pure enantiomers. As shown in Figure 2B, the pure enantiomers formed unbranched fibers, while the mixed enantiomers co-assembled into crystalline flake-like structure that was mechanically more robust than the enantiopure fibers. In addition to the peptides taking β configuration that can co-assemble with their enantiomers, the enantiomeric interactions of mixed enantiomers that take helix structure have also been studied. For example, the Nanda group found that mixing a collagen mimetic peptide (PPG)10 and its D-analog (DPDPG)10 drastically lowered the solubility, as they assembled into sheets and precipitated from the buffer solution, while in the same condition, individual enantiomer was soluble (Xu et al., 2013). Combining the results of experiments and computational simulation, the authors postulated that this helix peptide favored heterochiral association, since left- and right-handed molecular screws could interdigitate and pack more tightly (Figure 2C). In addition, phenol-soluble modulin α3 (PSMα3) and its D-analog, which take α-helical structure, can co-assemble into fibers with cross-α packing pattern supported by fiber diffraction data (Yao et al., 2019).

### Regulation of Chirality Effects on the Bioactivity of Peptide Assembly Structures

Introducing *D*-amino acids into self-assembled *L*-peptides is widely used to improve the enzymatic stability of their assembly structures, while it can also affect their biological functions. Chirality of amino acid residues/peptides can regulate the cell cytotoxicity of peptide assemblies. It was revealed that F*^*D*^*F could also self-assemble into nanotubes like its *L*-enantiomer, but the heterochirality completely alleviated its amyloid cytotoxicity (Kralj et al., 2020). Fibers formed by fatty chain-modified *L*-V_3_A_3_K_3_ showed higher cytotoxicity than the fibers formed by its *D*-analog, which was ascribed to the stronger affinity between the *L*-peptide and lipid (Sato et al., 2019). Ryu and coworkers designed a mitochondria-targeting peptide derivative Mito-FF, which was achieved by conjugating diphenylalanine with triphenyl phosphonium (TPP), a well-known mitochondria-targeting moiety. Mito-FF and its enantiomer Mito-*^*D*^*F*^*D*^*F co-assembled into nanofibers with diameter significantly larger than the nanofibers formed by Mito-FF. It was found that the co-assembled structure showed enhanced mitochondrial disruption, higher cellular cytotoxicity, and higher tumor inhibition due to its larger size (Jeena et al., 2019). Chirality of amino acid residues/peptides can affect the regulation of peptide assemblies on cell behaviors. Feng and coworkers constructed fibrillar hydrogels with opposite handedness through a series of phenylalanine derivative enantiomers and studied their regulatory effects on cell behaviors. The results suggested that left-handed helical nanofibers formed by *L*-enantiomer increased cell adhesion and proliferation, while right-handed helical nanofibers formed by *D*-enantiomer had the opposite effects (Liu et al., 2014). They also studied the difference of molecular and supramolecular chirality effects on cell differentiation and spreading, finding that amplification of chirality from chiral molecules to chiral assemblies dramatically increased the regulatory effect on cell behaviors by supramolecular helical handedness (Dou et al., 2019). In addition, stem cell lineage diversification was shown to be directed by the chirality of fibrillar matrix. Left-handed matrix formed by *L*-phenylalanine derivative was conductive for osteogenic lineage, while right-handed matrix formed by its *D*-analog was conductive for adipogenic lineage (Wei et al., 2019). These different regulatory effects on cells were suggested to be closely related to stereospecific interactions between peptide assemblies and proteins (Dou et al., 2019, 2020; Wei et al., 2019; Sun et al., 2021).

## Conclusion

In the process of investigating the chirality effects in peptide assemblies, most studies have focused on the impacts of amino acid chiral mutations on the assembly structure of peptides in the early days. In recent years, enantiomeric peptide interactions and constructing peptide assemblies with designed handedness have been paid much more attention. In this mini-review, we summarized the recent progresses in exploring the effects of chirality on the structure and bioactivity of peptide assemblies. In terms of structure, most studies focus on the effects of chirality alteration on the morphology, size, and secondary structure of peptide assemblies, while the molecular mechanisms for these effects are relatively less explored. It is worth noting that many researchers have combined Molecular Dynamics (MD) calculations with experimental data and successfully elucidated the molecular basis for the changes in the assembly structures of peptides caused by chirality conversion (Wang M. et al., 2017; Clover et al., 2020). Therefore, the combination of computational approaches and an arsenal of experimental techniques is supposed to be a useful tool to undercover the chirality effects in peptide assemblies on the molecular level. In terms of biological function, incorporating *D*-amino acids into self-assembled peptides was originally to enhance their resistance to enzymatic degradation. However, it can be seen from the above results that chirality is a key factor that can greatly change the morphology, size, and handedness of peptide assemblies and can even disrupt them. It has been demonstrated that these parameters are closely related to the bioactivity of peptide assemblies (Haass and Selkoe, 2007; Liu et al., 2018). In addition, due to the stereoselectivity of interactions between biomolecules, such as peptide–peptide interactions and peptide–lipid interactions (Ishigami et al., 2015; Chen et al., 2019), *D*-amino acid incorporation will affect the interactions between peptide self-assembled materials and their surrounding environment. As a result, *D*-amino acid substitution will inevitably affect the biological function of peptide assemblies due to its structural regulation on supramolecular structures and the chirality-dependent interactions between biomolecules. Therefore, the influence of chirality conversion on the biological performances of peptide assemblies should be comprehensively evaluated, not just from the aspect of enzyme stability.

## Author Contributions

All authors listed have made a substantial and intellectual contribution to the work and approved it for publication.

## Conflict of Interest

The authors declare that the research was conducted in the absence of any commercial or financial relationships that could be construed as a potential conflict of interest.
